# Elevated All-Cause Mortality among Overweight Older People: AI Predicts a High Normal Weight Is Optimal

**DOI:** 10.3390/geriatrics7030068

**Published:** 2022-06-16

**Authors:** Kei Nakajima, Mariko Yuno

**Affiliations:** 1 School of Nutrition and Dietetics, Faculty of Health and Social Services, Kanagawa University of Human Services, 1-10-1 Heisei-cho, Yokosuka 238-8522, Japan; yuno-mariko.5l3@mhlw.go.jp; 2 Department of Endocrinology and Diabetes, Saitama Medical Center, Saitama Medical University, 1981 Kamoda, Kawagoe 350-8550, Japan; 3 Department of Food and Nutrition, Faculty of Human Sciences and Design, Japan Women’s University, 2-8-1 Mejiro-dai, Bunkyo-ku, Tokyo 112-8681, Japan

**Keywords:** overweight, mortality, optimal, artificial intelligence, body mass index, elderly

## Abstract

It has been proposed that being overweight may provide an advantage with respect to mortality in older people, although this has not been investigated fully. Therefore, to confirm that and elucidate the underlying mechanism, we investigated mortality in older people using explainable artificial intelligence (AI) with the gradient-boosting algorithm XGboost. Baseline body mass indexes (BMIs) of 5699 people (79.3 ± 3.9 years) were evaluated to determine the relationship with all-cause mortality over eight years. In the unadjusted model, the first negative (protective) BMI range for mortality was 25.9–28.4 kg/m^2^. However, in the adjusted cross-validation model, this range was 22.7–23.6 kg/m^2^; the second and third negative BMI ranges were then 25.8–28.2 and 24.6–25.8 kg/m^2^, respectively. Conversely, the first advancing BMI range was 12.8–18.7 kg/m^2^, which did not vary across conditions with high feature importance. Actual and predicted mortality rates in participants aged <90 years showed a negative-linear or L-shaped relationship with BMI, whereas predicted mortality rates in men aged ≥90 years showed a blunt U-shaped relationship. In conclusion, AI predicted that being overweight may not be an optimal condition with regard to all-cause mortality in older adults. Instead, it may be that a high normal weight is optimal, though this may vary according to the age and sex.

## 1. Introduction

During recent decades, several studies have shown that, compared to a normal weight, being overweight or obese may confer an advantage in terms of reducing mortality in older patients, while being underweight (malnutrition) is a robust risk factor for increased mortality [1,2,3]. Yet, most studies demonstrating this relationship have evaluated older outpatients with fatal conditions such as heart failure, renal failure, and stroke, or older inpatients receiving routine treatments; this has been termed the obesity paradox [4,5,6]. Few studies have investigated this phenomenon in the general community-dwelling population of older people, although large proportions of older people develop latent comorbidities as they age.

In the past decade, artificial intelligence (AI), including machine learning and artificial neural networks, has been used to predict the incidence of various diseases and support their management [7,8].

Nevertheless, no study had yet investigated the relationship between mortality and body weight (assessed by the body mass index (BMI)) among older people living in the community using AI analysis. To that end, and to elucidate the underlying mechanism, we applied an explainable AI analysis system with higher prediction accuracy, for outcomes such as those of patients with colorectal cancer or heart failure, compared to a traditional analysis [9,10].

## 2. Methods

### 2.1. Study Design

To evaluate the relationships between baseline BMI and all-cause mortality in the general population of older patients, we conducted an eight-year retrospective cohort study. The ethics committee of Kanagawa University of Human Services approved the study protocol (approval number 17-26) and the study followed the ethical principles of the Declaration of Helsinki. Given that it was a retrospective study using anonymized data, the requirement for informed consent was waived. The study protocol was shared on the university’s homepage [11] and published in the public relations magazine of Yamato, Kanagawa Prefecture, Japan [12]. The details of the protocol are described elsewhere [13].

### 2.2. Participants, Measurements, and AI Analysis

Baseline clinical data for 5958 older people living in Yamato City were reviewed between April 2011 and March 2012. All participants had undergone an annual physical examination and blood tests and responded to questions prepared by medical personnel. A total of 259 subjects whose survival and data could not be confirmed throughout the eight years, because they left the city or for other reasons, were excluded. Therefore, 5699 subjects (79.3 ± 3.9 years, 2449 men and 3250 women) remained for analysis in the study.

To achieve a prediction with the highest possible accuracy for all-cause mortality, we used an AI analysis system (Prediction One [Sony Network Communications Inc., Tokyo, Japan]) [14] that implements an extreme gradient-boosting (XGboost) algorithm and calculates the feature importance, a method of explainable AI [15]. Feature importance reflects the relative contribution degree as a continuous value when the factor is considered in the tree algorithm. Cross-validation testing was also included in the AI analysis because it improves the general applicability and prevents overfitting [16,17]. The age, sex, physical activity, smoking status, pharmacotherapies (for hypertension, diabetes, dyslipidemia), cardiovascular disease history, and alcohol consumption were added as confounding factors to the prediction models. The model performance was evaluated by the area under the curve (AUC) values, obtained using receiver operating characteristic curve analysis.

## 3. Results

When we consider the baseline characteristics of the participants, all averages of the clinical parameters were near normal, as reported in a previous study [13]. The average participant ages were 79.3 ± 3.8 for men and 79.4 ± 4.0 for women. During the eight years of the study, 1413 (24.8%) participants died.

In the same period, 1632 (28.6%) participants developed overt disability (a long-term care level of ≥2). Overall, the mean follow-up time was 7.22 years.

Table 1 and Table 2 present different features’ importance for the contribution of BMI toward mortality. In the unadjusted no-cross-validation model (Table 1), the first negative (protective) range of BMI for mortality was 25.9–28.4 kg/m^2^, and the second and third ranges were 22.7–23.6 and 24.6–25.9 kg/m^2^, respectively. Conversely, the first positive (advancing) range of BMI for mortality was 12.8–18.7 kg/m^2^, with a relatively higher feature importance (0.087). In the cross-validation model with five divisions, unlike in the no-cross-validation model, the first negative range of BMI was 21.0–21.9 kg/m^2^, and the second and third ranges were 22.7–23.6 and 25.8–28.2 kg/m^2^, respectively. Similar to the no-cross-validation model, the first positive range of BMI was 12.8–18.7 kg/m^2^.

Table 2 shows the results obtained using the adjusted models. Regardless of cross-validation, age and sex (male) were the first and second positive contributors in the models with higher feature importance. In the no-cross-validation model, the first negative range of BMI was 22.7–23.6 kg/m^2^, and the second and third ranges were 25.9–28.4 and 24.6–25.9 kg/m^2^, respectively. Meanwhile, the first and second positive ranges of BMI were 12.8–18.7 and 23.6–24.6 kg/m^2^, respectively. The first to third negative ranges of BMI in the cross-validation model were nearly identical to those in the no-cross-validation model. The first and second positive ranges of BMI were likewise the same in the no-cross-validation model.

The total classification accuracies (AUCs) of the models were higher in the adjusted contribution models (73.7% and 69.6%) than in the unadjusted models (58.4% and 53.7%).

**Figure 1:**
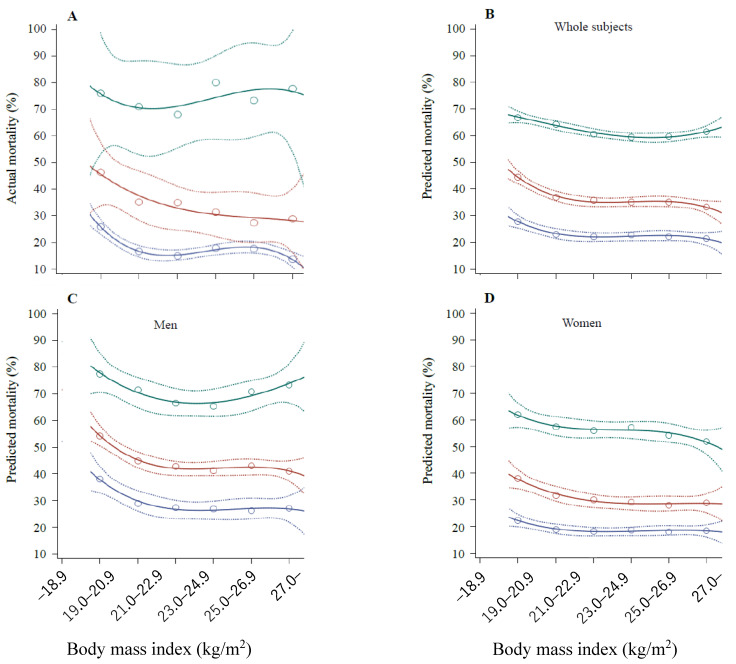
Mortality rates over the eight years according to BMI categories and age groups. (**A**) Actual mortality rates of all participants. This percentage was calculated as follows: number of deaths/number of baseline participants in each BMI category × 100. The numbers of participants in each BMI category were 364, 667, 833, 751, 500, and 338 in the 67–79-year-old category; 278, 468, 525, 424, 275, and 156 in the 80–89-year-old category; 25, 31, 25, 15, 15, and 9 in the 90–104-year-old category. (**B**) Predicted mortality rates of all participants based on the predicted mortality for individuals. The numbers of participants in each BMI category were the same as those mentioned in panel (**A**). (**C**) Predicted mortality rates in male participants based on the predicted mortality for individuals. The numbers of participants in each BMI category were 127, 275, 350, 373, 243, and 113 in the 67–79-year-old category; 111, 186, 231, 206, 132, and 55 in the 80–89-year-old category; 8, 15, 11, 4, 5, and 4 in the 90–104-year-old category. (**D**) Predicted mortality rates in female participants based on the predicted mortality for individuals. The circles and broken lines show the means and 95% CIs. The numbers of participants in each BMI category were 237, 392, 483, 378, 257, and 225 in the 67–79-year-old category; 167, 282, 294, 218, 143, and 101 in the 80–89-year-old category; 17, 16, 14, 11, 10, and 5 in the 90–104-year-old category. The lines and circles were made using SAS software.

Figure 1 depicts the actual mortality rates and averages for individuals as predicted through AI analysis during the eight years according to BMI categories and age groups. Due to the wide range of 95% confidence intervals (CIs), the actual mortality rates (Figure 1A) were not categorized according to the sex. Regardless of the actual and predicted rates and sexes, the mortality rates in participants aged <90 years showed a negative-linear or L-shaped relationship with BMI. However, the predicted mortality rates in male participants aged ≥90 years (Figure 1C), which were similar to the actual mortality rates and higher than those of other age groups, showed a blunt U-shaped relationship with BMI.

## 4. Discussion

This is the first study to address the relationship between the baseline BMI and mortality in the near future in older people using explainable AI. Explainable AI provides helpful information including the feature importance, reflecting the relative contribution degree to the outcome [15]. Although the age and sex (unmodifiable factors) were the strongest predictors of mortality (Table 2), our results demonstrated that BMI was the third contributor to mortality. The first positive (advancing) range of BMI for mortality, of 12.8–18.7 kg/m^2^, which usually reflects malnutrition, did not vary across the conditions, with a high feature importance regardless of cross-validation and covariate adjustment. Although the second positive range of BMI in the three models was 23.6–24.6 kg/m^2^, a high normal weight, the feature importance (0.020–0.026) in these ranges was small, at one-third to one-quarter of the first positive range of BMI of 12.8–18.7 kg/m^2^ (0.080–0.091; Table 1 and Table 2). This indicates that a low body weight is a persistent strong risk factor for increased mortality, which is consistent with the findings of previous studies [2,3,18].

The crude, no-cross-validation model revealed that the protective range of BMI for mortality was 25.9–28.4 kg/m^2^, implying that being overweight (obese for Japanese people) is an optimal condition that minimizes the all-cause mortality among older people. Figure 1 partially presents this simple unadjusted result, which is consistent with the results of recent studies using conventional statistical analyses [1,2,3,4,5,6]. However, in the cross-validation model, the orders of feature importance were different, indicating that a normal weight represents the first and being overweight (25.8–28.2 kg/m^2^) represents the third protective range for mortality. Regardless of cross-validation, the adjusted models revealed that the first protective range of BMI was 22.7–23.6 kg/m^2^, implying that a high normal weight may be the optimal condition to minimize all-cause mortality, while being overweight (or obese in Japan) is the second-leading condition to minimize mortality when covariates (confounding factors) are adjusted.

Given that the feature importance of the negative range of 22.7–23.6 kg/m^2^ BMI (0.032–0.049) was smaller than (almost half) that of the positive range of 12.8–18.7 kg/m^2^ BMI (0.080–0.091), and since the second positive range of BMI was 23.6–24.6 kg/m^2^ (a high normal weight), the overall relationship of a high normal weight or being overweight with mortality, if any, may not be strong, and it may be more complicated than the robust relationship between a low BMI and mortality in older people. This result may be consistent with the previous results of a study conducted on Japanese people [18].

Although common comorbidities of hypertension, diabetes, dyslipidemia, and cardiovascular disease were considered confounding factors in the models (Table 2), the cause of mortality and other concomitant comorbidities, such as cancers and infections, data for which were not available in this study, may influence the relationship between a normal-to-high BMI and mortality. Furthermore, considering that Japanese people have had the world’s greatest life expectancies in recent decades [19], the relationship between mortality rates and body weight might vary across different age groups and between men and women, even in their last stage of life.

According to Schooling et al. [20], obesity is associated with better outcomes in older people with a poor health status, which has been called the obesity paradox, now documented for several decades, but it is associated with a worse condition in those with an initially good health status. In this study, most of the subjects were apparently healthy people who had not been admitted to a hospital or nursing home at the beginning of the study period. That factor may have affected the observed outcomes

In this study, the relationship between the baseline BMI and mortality appeared to be negative-linear or L-shaped in participants in their 80s, which corresponds to the average Japanese life span, although no such relationship was observed in other age groups. Therefore, being overweight or obese might be advantageous for older people, positively impacting their longevity, mostly for older people with a poor health status. However, gaining weight to lower the risk of mortality in the near future might be unnecessary in older people if they have a high normal weight; this applies at ages both above and below the average life expectancy of the population.

In bringing this article to a close, methodological limitations such as the retrospective design, possible selection bias, and use of BMI as the obesity index [4] should be noted. As the BMI does not distinguish muscle from fat, additional measurements of the body composition and fat distribution [5], as well as fitness [6], may be useful for determining the health status of older people. In addition, the follow-up period of 7.22 years may be short to evaluate the relationship between BMI and mortality. As such, a long-term study including various obesity indices is needed to better evaluate the relationship between obesity and mortality in the elderly. Alternatively, further studies with different modes of extreme gradient boosting, such as decision tree and random forest, and populations with varying life expectancies, are warranted to confirm the results of the present study and underlying mechanisms.

## 5. Conclusions

Our results from an explainable AI analysis suggest that a low body weight is a prominent risk factor, but that being overweight or obese may not be the optimal condition to minimize all-cause mortality among community-dwelling older Japanese people. It is suggested that a high normal weight may be the optimal condition, though the suitability of this varies according to the age, sex, and comorbidities.

## Figures and Tables

**Table 1:** Crude (unadjusted) contributions of the ranges of BMI toward all-cause mortality.

Contribution Order	Range of BMI (kg/m^2^)	Feature Importance 1 *	Feature Importance 2 **
No-cross-validation model			
Negative (protective)			0.087
1	25.9–28.4	0.033	
2	22.7–23.6	0.032	
3	24.6–25.9	0.027	
Positive (advancing)			
1	12.8–18.7	0.087	
2	23.6–24.6	0.026	
3	18.7–20.0	0.017	
Total classification accuracy (AUC) 58.4%			
Cross-validation model			
Negative (protective)			0.101
1	21.0–21.9	0.060	
2	22.7–23.6	0.049	
3	25.8–28.2	0.039	
Positive (advancing)			
1	12.8–18.7	0.087	
2	18.7–20.0	0.019	
3	20.0–21.0	0.014	
Total classification accuracy (AUC) 53.7%			

Feature importance reflects the contribution degree as a continuous value. * Feature importance in the detail range of BMI. ** Feature importance of BMI itself. Cross-validation was automatically performed after the total data were divided into five divisions. BMI: body mass index.

**Table 2:** Adjusted contributions of the ranges of BMI toward all-cause mortality.

Contribution Order	Range of BMI (kg/m^2^)	Feature Importance 1 *	Feature Importance 2 **	Feature Importance 3 ***
No-cross-validation model				
Negative (protective)			0.080	Age: 0.192 Sex (men): 0.133
1	22.7–23.6	0.037		
2	25.9–28.4	0.029		
3	24.6–25.9	0.023		
Positive (advancing)				
1	12.8–18.7	0.080		
2	23.6–24.6	0.020		
3	20.0–21.0	0.007		
Total classification accuracy (AUC) 73.7%				
Cross-validation model				
Negative (protective)			0.099	Age: 0.253 Sex (men): 0.129
1	22.7–23.6	0.046		
2	25.8–28.2	0.035		
3	24.6–25.8	0.025		
Positive (advancing)				
1	12.8–18.7	0.091		
2	23.6–24.6	0.024		
3	18.7–20.0	0.018		
Total classification accuracy (AUC) 69.6%				

Feature importance reflects the contribution degree as a continuous value. * Feature importance in the detail range of BMI. ** Feature importance of BMI itself. *** Feature importance of age and sex, which were greater than that of BMI. Cross-validation was automatically performed after dividing the total data into five divisions. In the no-cross-validation model considering confounding factors, the contribution order of feature importance was age, sex, BMI, physical activity, pharmacotherapy for dyslipidemia, smoking status, pharmacotherapy for hypertension, pharmacotherapy for diabetes, cardiovascular disease history, and alcohol consumption. In the cross-validation model considering confounding factors, the contribution order of feature importance was age, sex, BMI, smoking status, physical activity, cardiovascular disease history, pharmacotherapy for dyslipidemia, diabetes, hypertension, and alcohol consumption. Both models used the 10 variables mentioned above when they predicted the mortality from contributing factors and calculated the feature importance. BMI: body mass index. Physical activity: performing or not performing exercise regularly, with those who are active defined as performing ≥30 min of exercise at least twice a week.

## Data Availability

Not applicable.

## References

[1] Flegal K.M., Kit B.K., Orpana H., Graubard B.I. (2013). Association of all-cause mortality with overweight and obesity using standard body mass index categories: A systematic review and meta-analysis. JAMA.

[2] Lee J.S., Auyeung T.W., Chau P.P., Hui E., Chan F., Chi I., Woo J. (2014). Obesity can benefit survival-a 9-year prospective study in 1614 Chinese nursing home residents. J. Am. Med. Dir. Assoc..

[3] Veronese N., Cereda E., Solmi M., Fowler S.A., Manzato E., Maggi S., Manu P., Abe E., Hayashi K., Allard J.P. (2015). Inverse relationship between body mass index and mortality in older nursing home residents: A meta-analysis of 19,538 elderly subjects. Obes. Rev..

[4] Karampela I., Chrysanthopoulou E., Christodoulatos G.S., Dalamaga M. (2020). Is There an Obesity Paradox in Critical Illness? Epidemiologic and Metabolic Considerations. Curr. Obes. Rep..

[5] Bosello O., Vanzo A. (2021). Obesity paradox and aging. Eat Weight Disord..

[6] Khan A., Van Iterson E.H., Laffin L.J. (2021). The obesity paradox in heart failure: What is the role of cardiorespiratory fitness?. Cleve. Clin. J. Med..

[7] Hasanzad M., Aghaei Meybodi H.R., Sarhangi N., Larijani B. (2022). Artificial intelligence perspective in the future of endocrine diseases. J. Diabetes Metab. Disord..

[8] Corti C., Cobanaj M., Marian F., Dee E.C., Lloyd M.R., Marcu S., Dombrovschi A., Biondetti G.P., Batalini F., Celi L.A. (2022). Artificial intelligence for prediction of treatment outcomes in breast cancer: Systematic review of design, reporting standards, and bias. Cancer Treat. Rev..

[9] Antonelli G., Badalamenti M., Hassan C., Repici A. (2021). Impact of artificial intelligence on colorectal polyp detection. Best Pract. Res. Clin. Gastroenterol..

[10] Kodera S., Akazawa H., Morita H., Komuro I. (2022). Prospects for cardiovascular medicine using artificial intelligence. J. Cardiol..

[11] https://www.kuhs.ac.jp/research/news/details_00248.htmlYamato.

[12] http://www.city.yamato.lg.jp/web/kouhou/n20181218-2.html.

[13] Nakajima K., Yuno M., Tanaka K., Nakamura T. (2022). High Aspartate Aminotransferase/Alanine Aminotransferase Ratio May Be Associated with All-Cause Mortality in the Elderly: A Retrospective Cohort Study Using Artificial Intelligence and Conventional Analysis. Healthcare.

[14] (2022). Sony Network Communications, Prediction One. https://predictionone.sony.biz.

[15] Casola S. (2021). What is Explainable Artificial Intelligence, and Why Is It Important for Predictive Models?. https://www.explorium.ai/blog/what-is-explainable-artificial-intelligence-and-why-is-it-important-for-predictive-models/.

[16] Gupta A., Kulkarni M., Mukherjee A. (2021). Accurate prediction of B-form/A-form DNA conformation propensity from primary sequence: A machine learning and free energy handshake. Patterns.

[17] Reps J.M., Ryan P., Rijnbeek P.R. (2021). Investigating the impact of development and internal validation design when training prognostic models using a retrospective cohort in big US observational healthcare data. BMJ Open.

[18] Hozawa A., Hirata T., Yatsuya H., Murakami Y., Kuriyama S., Tsuji I., Sugiyama D., Satoh A., Tanaka-Mizuno S., Miura K. (2019). Association Between Body Mass Index and All-Cause Death in Japanese Population: Pooled Individual Participant Data Analysis of 13 Cohort Studies. J. Epidemiol..

[19] Tsugane S. (2021). Why has Japan become the world’s most long-lived country: Insights from a food and nutrition perspective. Eur. J. Clin. Nutr..

[20] Schooling C.M., Lam T.H., Li Z.B., Ho S.Y., Chan W.M., Ho K.S., Tham M.K., Cowling B.J., Leung G.M. (2006). Obesity, physical activity, and mortality in a prospective chinese elderly cohort. Arch. Intern. Med..

